# A new genus and new species of Agathotanaidae (Crustacea, Tanaidacea) from West Australia

**DOI:** 10.3897/zookeys.243.3408

**Published:** 2012-11-16

**Authors:** Piotr Jóźwiak, Aleksandra Jakiel

**Affiliations:** 1 Laboratory of Polar Biology and Oceanobiology, University of Łódź, Banacha 12/16, Łódź, Poland

**Keywords:** *Bunburia*, NW Australia, Tanaidacea, Agathotanaidae

## Abstract

A new genus of Tanaidacea – *Bunburia*,collected from the region of Ningaloo in the vicinity of Bunbury (Western Australia), is erected to accommodate the new species – *Bunburia prima* **sp. n.** This genus is classified in the family Agathotanaidae and it can be distinguished from the other members of the family by having a combination of antennulae covered with minute setae, reduced uropods and unusual setation of the propodus of pereopods 4 to 6. *Bunburia prima* is the second species of Agathotanaidae known so far from Australia.

## Introduction

The family Agathotanaidae erected by Lang (1971a) is represented by 41 species in four genera, namely *Agathotanais* Hansen, 1913, *Metagathotanais* Bird and Holdich, 1988, *Paragathotanais* Lang, 1971 and *Paranarthrura* Hansen, 1913 (Anderson 2012). The fifth genus, *Paranarthrurella* Lang, 1971, which had been previously considered as a member of this family (Larsen and Wilson 2002, Larsen 2005) was recently removed from Agathotanaidae (Jóźwiak et al. 2009).

The diagnosis of Agathotanaidae, modified by Larsen (2005), includes mandibles having a pointed or reduced molar, the absence of pleopods in the females and the uropod endopod that consists of one or two articles. The uropod exopods of Agathotanaidae is reduced to a blunt spur and only the presence of distal and middle setae suggests it is a fused exopod rather than a basis process. Another character that Larsen (op. cit.) has pinpointed as diagnostic for Agathotanaidae is cheliped attached directly to the cephalothorax or via pseudocoxa, however it was later questioned by Larsen (2007) and Bird (2010).

The genus *Agathotanais* is distinguishable from the other agathotanaid genera by its finely setulose body, 3-articled antennulae, reduced antennae, cheliped attached directly to the cephalothorax and uropodal endopod fused to the basis (Hansen 1913, Larsen 1999, 2005). *Metagathotanais* is the genus with strongly-reduced uropods (endopod fused with basis), but in contrast to *Agathotanais* it has fully-developed antennae that consist of six articles and 4-articled antennulae (Guerrero-Kommritz 2003). An unique feature of *Metagathotanais* is the fusion of the pleonites with the pleotelson in females, although the males retain complete pleonite segmentation. Members of *Paragathotanais* have both antennulae and antennae well-developed and the pleon with five distinct pleonites, while the uropods endopod is separated from the basis (Lang 1971b, Bird and Holdich 1988, Larsen 2005). *Paranarthrura* is the only genus of the family with uropod supported by a visible projection. The number of articles in the uropodal endopod of *Paranarthrura* can be either one or two.

The collection of Tanaidacea from the shelf and continental margin of Western Australia (WA), taken from on board the FRV *Southern Surveyor* in 2005 and 2007 has represented close to three hundred species new for science (Poore et al. in press). About 60% of the taxa are represented by single or few specimens only, while 82% of the taxa occurred just twice in the series of over two hundred samples. This scarcity of the material, together with the minute size of the specimens, which are often no longer than 1 mm, impede the formal taxonomical descriptions of the species.

One of the few species represented by more than one specimen was found to be a representative of a new genus of the family Agathotanaidae. The present paper presents the formal description of this species and the definition of the new genus that has been erected to accommodate it.

## Material and methods

The material was collected in 2005 during the voyage of the FRV *Southern Surveyor* organized under the aegis of CSIRO (*Commonwealth Scientific and Industrial Research Organization)*. The 14 specimens studied by us were recorded at ten of two hundred grab samples taken along the west coast of Australia from Dampier in the north to Albany in the south (from 21.0084°S, 114.381°E to 35.384°S, 118.316°E).

Appendages were dissected using chemically-sharpened tungsten-wire needles, stained with chlorazol black and mounted in glycerine. Drawings were prepared using a microscope combined with a *camera lucida* and redrawn on a digital tablet as proposed by Coleman (2003). The morphological terminology follows that proposed by Błażewicz-Paszkowycz and Bamber (2007). The body-length to width ratio was calculated using measurements from tip of the rostrum to end of pleotelson and of the widest part of cephalothorax. The ratio of particular articles was measured along their central axis. Abbreviations used in the morphological description: A1 – antennule, A2 – antenna, Mxp – maxilliped, P1-P6 – pereopods from first to sixth pair respectively.

The type material is deposited at Museum Victoria, Melbourne.

## Systematics

### Order Tanaidacea Dana, 1849

Suborder Tanaidomorpha Sieg, 1980

Family Agathotanaidae Lang, 1971

#### Bunburia gen. n.

urn:lsid:zoobank.org:act:70EF00B3-B23A-4C38-B552-1E4FBDBE6192

http://species-id.net/wiki/Bunburia

##### Diagnosis.

Pleon with five free pleonites; antennulae 4-articled, with first article covered by numerous minute setae; antenna 6-articled, article 3 with dense setation; mandibles molar reduced; labium without outer or medial, setose process; maxillipedal bases unfused distally, endites unfused; epignath elongated and naked; cheliped sclerites unfused ventromedially, carpus stout (1.4 times as long as wide), chela with keel; pereopods with coxa; P1 propodus with elongate ventral seta, P1-P3 merus with long serrated seta, P4-P6 propodus with two long, serrated setae ventrodistally and three short setae dorsodistally, P5 and P6 with propodus shorter than carpus, dactylus/unguis of these pereopods setulated ventrally; ischium of all pereopods with only one seta; pleopods absent in female and well developed in male; uropod short, not projecting beyond pleotelson, with basis terminated with small projection, endopod short, one-articled.

##### Type species.

*Bunburia prima* sp. n. *–* by monotypy.

##### Etymology.

The name refers to Bunbury, a port city near the type locality of *Bunburia prima* sp. n.

##### Remarks.

At first glance *Bunburia* gen. n., with its short uropods that not protrude the pleotelson, resembles the members of *Paragathotanais*. The new genus can be distinguish however from *Paragathotanais* by presence of dense setationon the proximal article of the antennulae and the fourth article of the antennae and by unusual chetotaxy of propodus of last three pereopods, which consists of three short setae dorsodistally and two long setae ventrodistally. The setation of propodus P4-P6 is variable in members of *Paragathotanais*. For example *Paragathotanais abyssorum* Larsen, 2007, *Paragathotanais insolitus* Guerrero-Kommritz, 2003 and *Paragathotanais ipy* Jóźwiak i Błażewicz-Paszkowycz, 2011 have three long, distal setae, while five other species: *Paragathotanais gracilis* Bird and Holdich, 1988, *Paragathotanais nanus* Bird and Holdich, 1988, *Paragathotanais robustus* Bird and Holdich, 1988, *Paragathotanais typicus* Lang, 1971 and *Paragathotanais vikingus* Bird, 2010 have three long setae in propodus of pereopods 4 and 5, but four setae in pereopod 6. Another species – *Paragathotanais macrocephalus* Kudinova-Pasternak, 1986 lack of setae on propodus of pereopod 4, but it has three setae in pereopod 6. In *Paragathotanais medius* Larsen, 2002 there are four long, distal setae at propodus of P4-P6. Beside the pereopods setation *Bunburia* can be separated from *Paragathotanais* by lack of medial process on the labium and bases of maxilliped unfused distally.

Larsen (2007) has pointed out that size of uropods and theirs position on the pleotelson distinguish *Paragathotanais* from *Paranarthrura*. *Bunburia* gen. n., with uropods similar to those observed in *Paragathotanais*, can be distinguished from *Paranarthrura* by short uropods, that are not reaching over pleotelson and are inserted more ventrally. The 4-articled antennula and the 6-articled antenna distinguish *Bunburia* from *Agathotanais*, that has 3-articled antennula and antenna reduced to one short article (Larsen 1999, 2005). An evident is also the difference between females of *Bunburia* and *Metagathotanais*, which have all pleonites fused with pleotelson (Bird and Holdich 1988, Guerrero-Kommritz 2003). Males of *Metagathotanais* have pleotelson with five distinct pleonites, but they differs from males of *Bunburia* in propodus P4-P6 chetotaxy. In *Metagathotanais insulcatus* Bird and Holdich, 1988 propodus of these pereopods bears one short and three long setae distally and in *Metagathotanais loerzae* Guerrero-Kommritz, 2003 there are only three long setae.

*Bunburia* represented by only one species is the second taxon of Agathotanaidae known so far from Australia, after *Agathotanais spinipoda* Larsen, 1999.

#### Bunburia prima sp. n.

urn:lsid:zoobank.org:act:ECDC9C27-D624-479B-9276-6E13A322B702

http://species-id.net/wiki/Bunburia_prima

**Figure 1.**
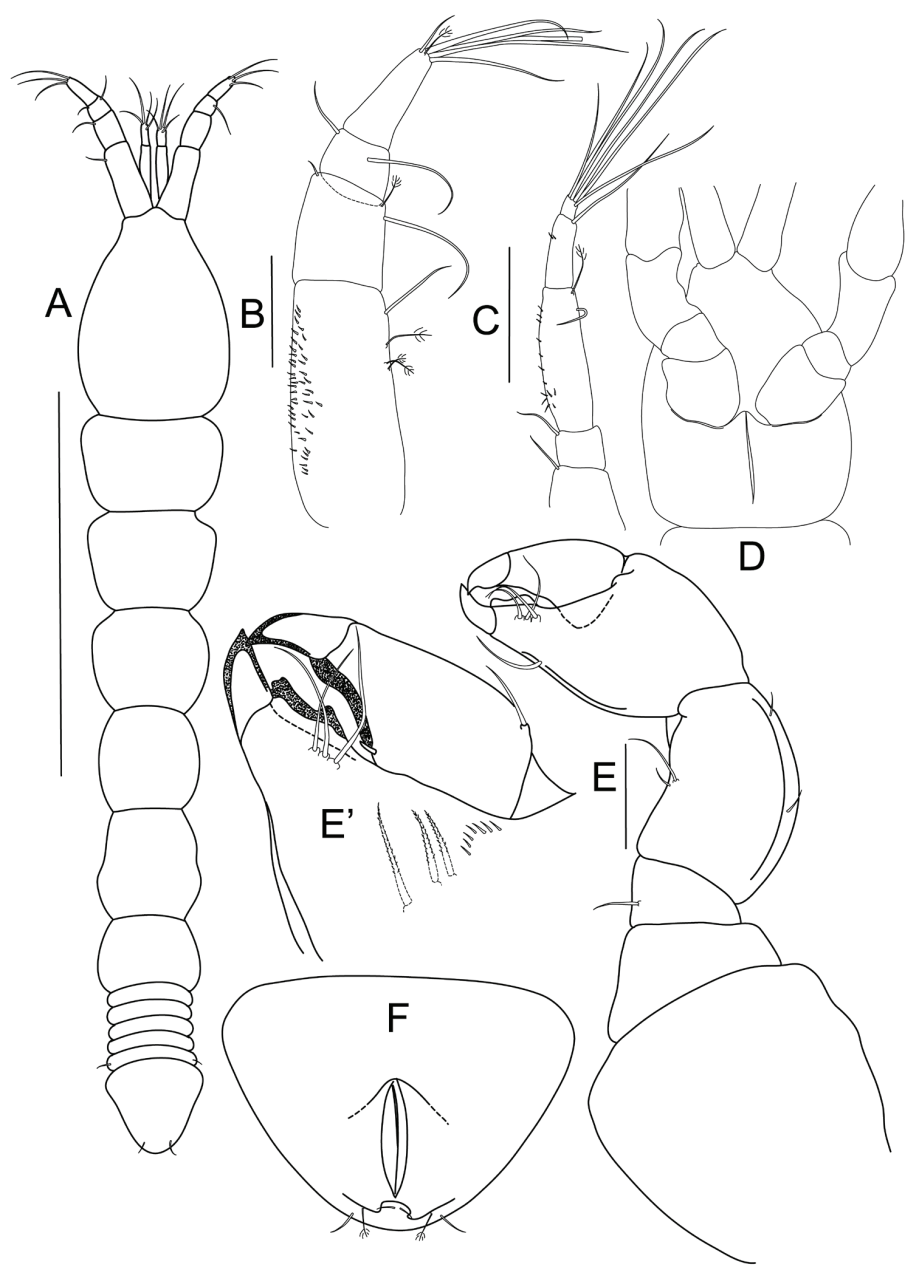
*Bunburia prima* sp. n., holotype female. **A** body, dorsal view; paratype female **B** antennule **C** antenna (proximal article not shown) **D** cephalothorax, ventral view **E** cheliped **E’** details of fixed finger and dactylus **F** pleotelson, ventral view. Scale lines = 1 mm for **A** and 0.1 mm for **B–E**.

Figs 1 [Fig F2] [Fig F3] 4

##### Etymology.

The Latin ordinal number ‘*prima*’ denotes the fact that the species described herein is the first member of genus *Bunburia*.

##### Material examined.

Holotype, non-ovigerous female, J62967, 2.5 mm long, St. SS07/2005, 153,Bunbury, 33.0003°S, 114.579°E, depth 399 m, 07 August 2005.

##### Paratypes.

1 female dissected on slides, J62968, St. SS07/2005, 152, Bunbury, 32.9987°S, 114.576°E, depth 417 m, 2005.

1 male partially dissected, J62969, St. SS07/2005, 85, Zyutdorp, 27.1676°S, 112.778°E, depth 375 m, 29 July 2005.

1 specimen, J62974, St. SS07/2005, 8, Ningaloo, 22.0796°S, 113.797°E, depth 205 m, 2005; 1 specimen, J62973, St. SS07/2005, 23, Ningaloo, 22.0629°S, 113.723°E, depth 715 m, 2005; 2 specimens, J62971, St. SS07/2005, 24, 22.0631°S, 113.724°E, depth 713 m, 2005; 1 specimen, J62972, St. SS07/2005, 68, Point Cloates, 22.859°S, 113.328°E, depth 448 m, 2005; 1 specimen, J62970, St. SS07/2005, 75, Carnarvon, 24.5875°S, 112.253°E, depth 405 m, 2005; 2 specimens, J63690, St. SS07/2005, 76, Carnarvon, 24.5863°S, 112.254°E, depth 405 m, 2005; 3 specimens, J62975, St. SS07/2005, 126, Jurian Bay, 29.8604°S, 114.372°E, depth 499 m, 2005.

##### Type locality.

near Bunbury, 33.0003°S, 114.579°E, depth 399 m.

##### Diagnosis.

as for thegenus.

##### Description of female.

Habitus (Figs 1A, F): body 2.5 mm long, 6.2 times as long as wide. Carapace 23% of total body length, 1.5 times as long as wide. Length/width ratios of pereonites 1 to 6: 0.7, 0.8, 0.9, 1.0, 1.1 and 0.7 respectively. Pleon about 18% of total body length; pleonites equal in length; fifth pleonite with lateral simple seta. Pleotelson (Fig. 1F) with a pair of bipinnate setae and two pairs of simple setae distally.

Antennule (Fig. 1B) 4-articled; article 1 longest, with three bipinnate setae and one simple seta on outer margin, inner margin covered with numerous minute setae; article 2 about 0.4 times as long as article 1, with one bipinnate and two simple setae distally; article 3 trapezoidal, wider than long, with two distal setae; article 4 twice as long as article 3, distally with one bipinnate, one short simple, five long simple setae and one aesthetasc.

Antenna (Fig. 1C) 6-articled; article 1 broken; article 2 with one simple seta distally; article 3 square, with one simple, distal seta; article 4 longest, four times as long as article 3, with one simple and one bipinnate setae distally and row of small spines laterally; article 5 half as long as article 4, with minute lateral setation and one long seta distally; last article very short, distally with six long setae.

**Figure 2. F2:**
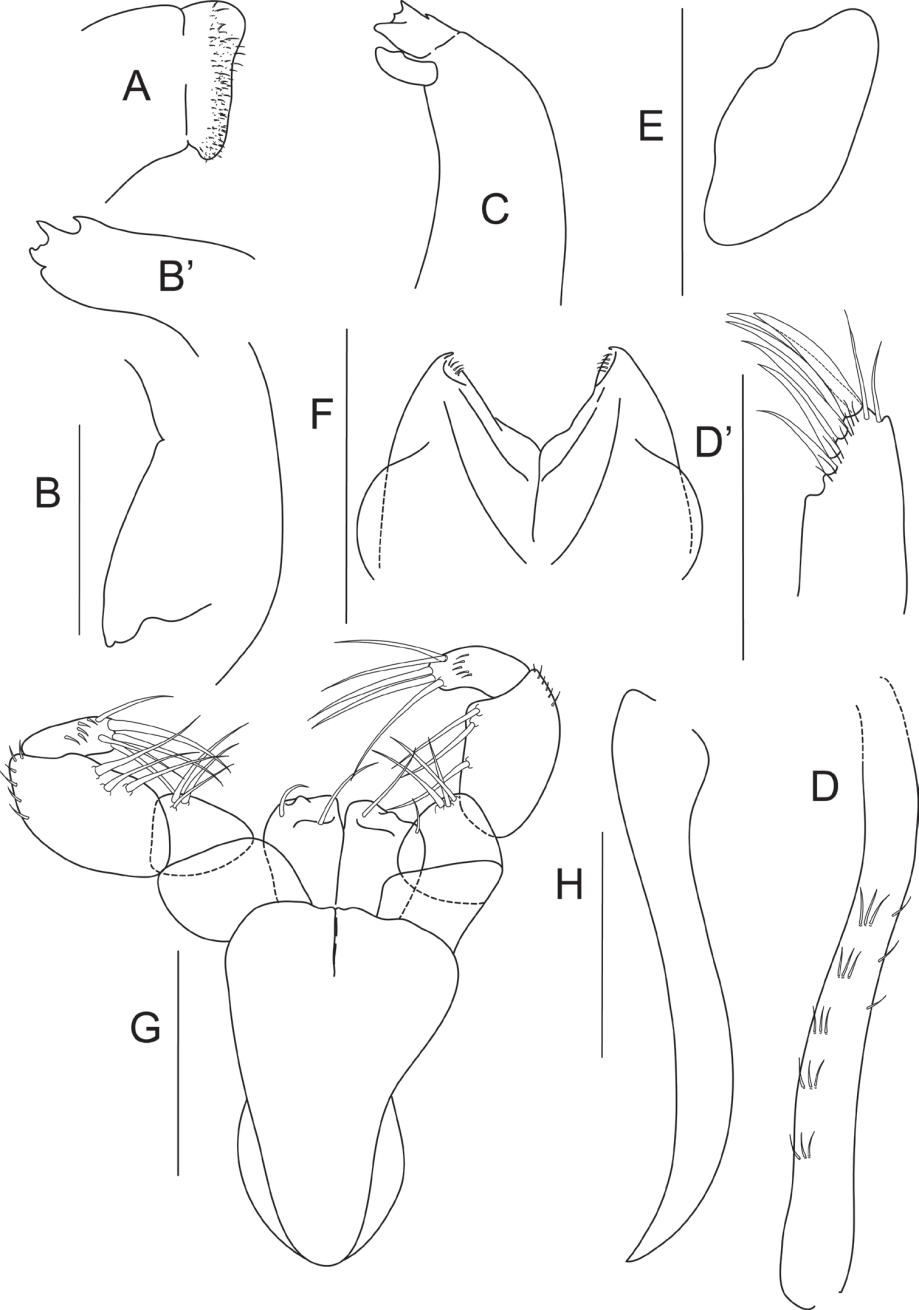
*Bunburia prima* sp. n., paratype female. **A** labrum **B** mandible molar **B’** incisor of right mandible **C** left mandible **D** maxillule endite **D** details of distal part of maxillule **E** maxilla **F** labium **G** maxilliped **H** epignath. Scale line = 0.1 mm.

Mouthparts: labrum (Fig. 2A) covered with dense setation; mandibles (Figs 2B, C) molar bent downward and tapering distally; right mandible incisor with four denticulations, left mandible with four denticulations on incisor and small lacinia mobilis with dorsal tooth. Maxillule (Figs 2D, D’) ventrally with combs of short, simple setae, distally with six spines, two simple setae and minute setation, palp lost during dissection; maxilla (Fig. 2E) ovate. Labium (Fig. 2F) bilobed, with minute setation distally. Maxilliped (Fig. 2G) bases unfused distally, endites with pair of distal, simple setae and one tubercle; palp article 1 naked; article 2 with three inner setae; article 3 with three setae on inner margin, outer margin setulated; last article with five long spiniform setae and minute setation. Epignath (Fig. 2H) elongated, strap-like and naked.

Cheliped (Figs 1D–E’) pseudocoxa massive, about as long as wide, naked, incompletely fused on midline of cephalothorax ventrum (Fig. 1D); basis trapezoidal and naked; merus triangular, with one seta ventrally; carpus 1.4 times as long as wide, with pair of setae on both dorsal and ventral margins; chela larger than carpus, propodus with one seta on ventral margin, inner comb of three serrated setae and row of minute spines; fixed finger with three setae on inner margin, and well calcified, inner teeth, ventrally with keel; dactylus with one small spiniform seta on inner margin and one seta dorsally.

**Figure 3. F3:**
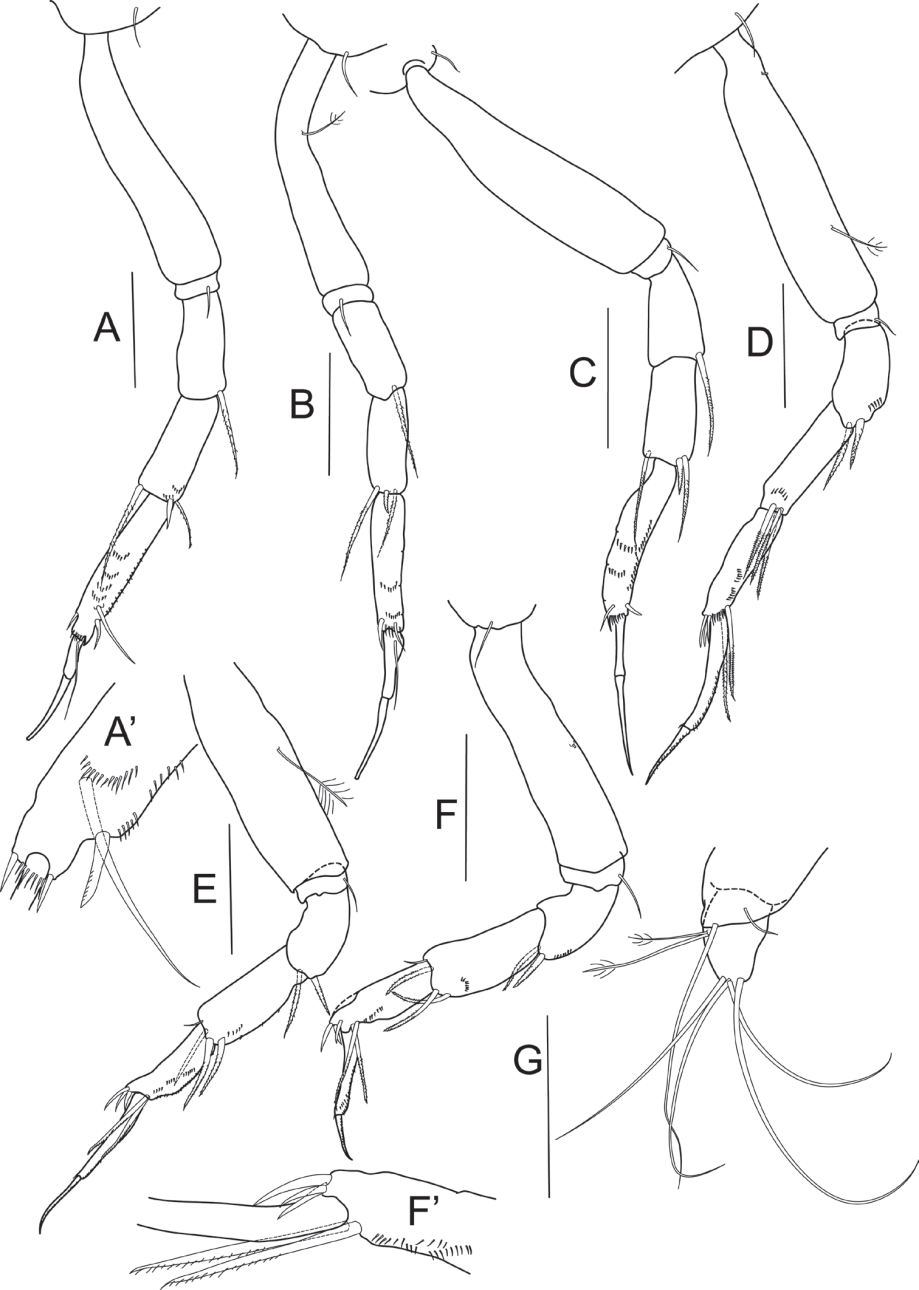
*Bunburia prima* sp. n., paratype female. **A** pereopod 1 **A’** detailes of P1 propodus **B** pereopod 2 **C** pereopod 3 **D** pereopod 4 **E** pereopod 5 **F** pereopod 6 **F’** details of P6 propodus **G** uropod. Scale line = 0.1 mm.

Pereopod 1 (Figs 3A, A’) coxa with seta; basis four times as long as wide, naked; ischium with simple seta; merus with one serrated seta; carpus as long as merus, with two serrated setae and one spiniform seta distally; propodus elongate, 1.5 times as long as merus, with three spiniform setae distally; dactylus 0.6 times as long as unguis, both together longer than carpus.

Pereopod 2 (Fig. 3B) coxa with simple seta; basis five times as long as wide, with one bipinnate seta; ischium with single seta; merus with one serrated seta; carpus as long as merus, with two serrated setae and one spiniform seta distally; propodus elongate, 1.2 times as long as merus, with two spiniform setae distally, combs of small spines present; dactylus little shorter than unguis, with proximal seta.

Pereopod 3 (Fig. 3C) coxa with simple seta; basis four times as long as wide; ischium with one seta; merus with serrated seta ventrodistally; carpus longer than merus, with one spiniform and two serrated setae distally; propodus elongate, almost twice as long as merus, with one spiniform seta ventrally, combs of minute spines present; dactylus 0.6 times as long as unguis.

Pereopod 4 (Fig. 3D) coxa with one seta; basis 4.6 times as long as wide, with one bipinnate seta ventrally; ischium with single seta; merus with two serrated setae distally and row of minute setation; carpus longer than merus, with three serrated, strong setae ventrally, minute setation present on ventral margin; propodus little longer than carpus, with two serrated and three spiniform setae distally, ventral margin with rows of minute setation; dactylus twice as long as unguis, with numerous minute spines ventrally.

Pereopod 5 (Fig. 3E) basis with one bipinnate seta; ischium with single seta; merus with two serrated setae ventrally; carpus longer than merus, with one simple and three serrated setae distally, minute setation on ventral margin; propodus clearly shorter than carpus, with two serrated long setae and three short spiniform setae, ventrally with rows of minute setation; dactylus almost twice as long as unguis, dorsal margin with minute setation.

Pereopod 6 (Figs 3F, F’) similar to pereopod 5, but dactylus/unguis slightly shorter.

Pleopods absent.

Uropod (Fig. 3G) basis with small projection bearing one long and one short, simple distal setae; endopod one-articled, as long as basal article, with four long, simple setae distally and two bipinnate setae at midlength of article.

**Figure 4.**
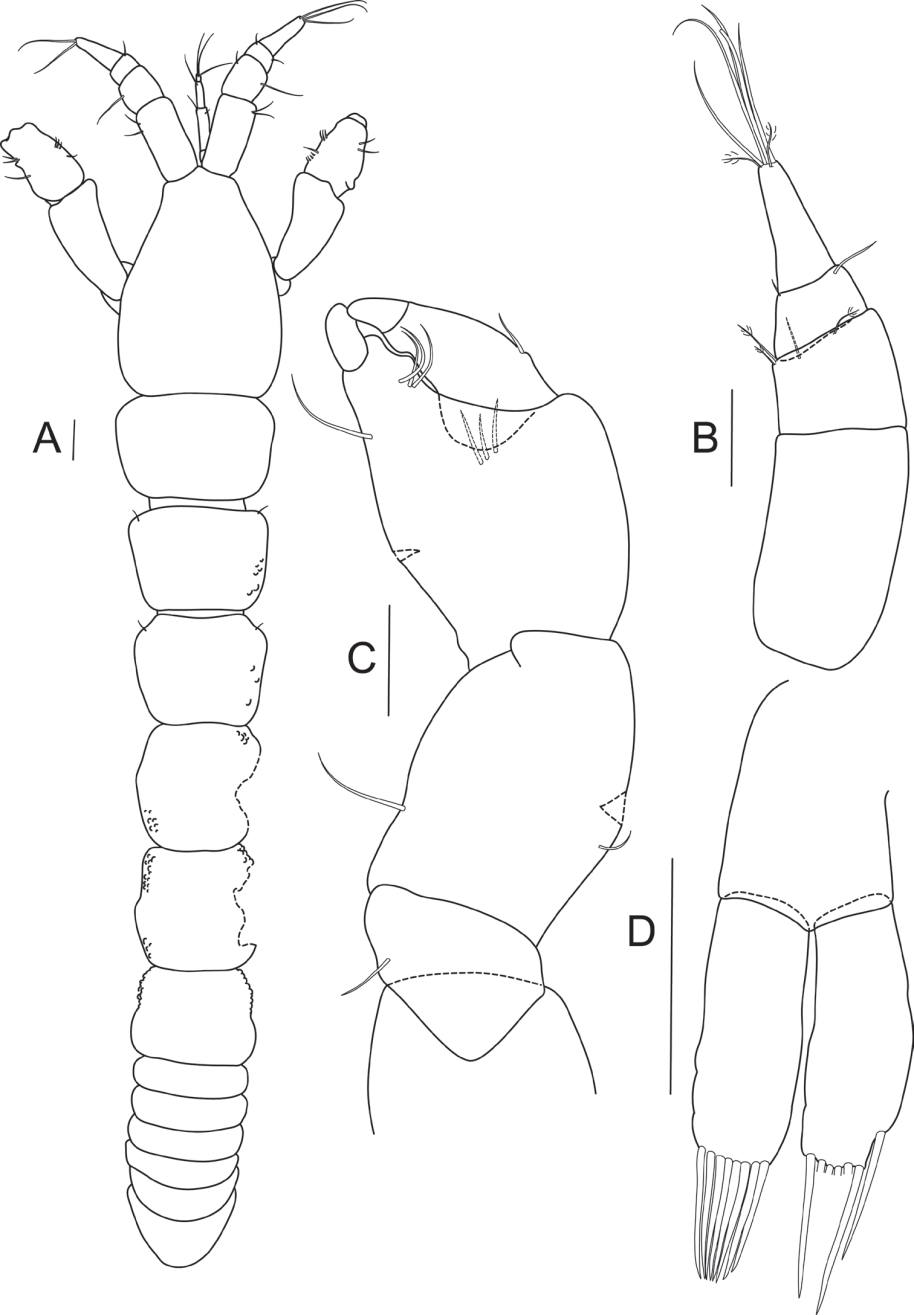
*Bunburia prima* sp. n., allotype male. **A** body, dorsal view **B** antennule **C** cheliped **D** pleopod. Scale line = 0.1 mm.

Male. Habitus (Fig. 4A): body 2.7 mm long, 6.7 times as long as wide. Carapace 20% of total body length, about 1.5 times as long as wide. Lateral margins of pereonites covered by small papillae; length/width ratios of pereonites 1 to 6: 0.7, 0.8, 0.8, 1.0, 1.0 and 0.8 respectively. Pleon about 18% of total body length; pleonites equal in length.

Antennule (Fig. 4B) stouter than that of female; 4-articled; article 1 longest and naked, 1.8 times as long as wide; article 2 0.4 times as long as first article, with four bipinnate setae distally; third article trapezoidal, about half as long as second article, with one outer and one inner simple setae; last article elongated, about half as long as first article; terminally with two bipinnate and five long, simple setae.

Cheliped (Fig. 4C) similar to that of female; merus with single seta; carpus stout – 1.3 times as long as wide, with single outer seta and pair of ventral setae (in figure only one). Propodus about as long as wide, with three setae near dactylus insertion, fixed finger with one ventral seta and three setae on inner (cutting) margin, ventral margin with keel. Dactylus as long as propodus, with single outer seta.

Pleopods (Fig. 4D) basis 0.8 times as long as each ramus, naked. Rami subequal, exopod terminating in eight strong, simple setae, endopod with one seta subdistally and six setae distally.

##### Distribution.

The species is known from Western Australia and was recorded between Ningaloo and Bunbury City in a depth range from 205 to 715 m.

## Supplementary Material

XML Treatment for Bunburia

XML Treatment for Bunburia prima
